# Evaluation of Retention Force Between PEEK Posts with Different Surface Treatments and Resin Composites for Core Build-Up by a Pull-Out Test—Effect of Thermal Cycling

**DOI:** 10.3390/ma19132694

**Published:** 2026-06-23

**Authors:** Masaaki Kasahara, Tomoko Someya, Hiroki Kagoura, Masayuki Hattori

**Affiliations:** Department of Dental Materials Science, Tokyo Dental College, Tokyo 101-006, Japankagourahiroki@tdc.ac.jp (H.K.); hattori@tdc.ac.jp (M.H.)

**Keywords:** polyetheretherketone (PEEK), resin composite for core build-up, surface treatment, retention force, thermal cycling, surface engineering

## Abstract

This study evaluated the retention force between milled polyetheretherketone (PEEK) posts with different surface treatments and resin composites for core build-up, and the effect of thermal cycling on the retention force. Four groups of PEEK posts were prepared: untreated group (NT), mechanically treated group with sandblasting (SB), chemically treated group with primer application (AD), and a group combining mechanical and chemical treatments (SB+AD). Pull-out tests were conducted on these groups. The specimens were divided into two subgroups: one stored in a humid environment at 37 °C for one week (TC0) and the other subjected to 10,000 cycles of thermal cycling between 5 °C and 55 °C (TC10,000). Data were analyzed using two-way ANOVA and Tukey’s test. Additionally, the effect of thermal cycling on each group was examined using Student’s *t*-test. Both surface treatment and thermal cycling factors had statistically significant effects on retention force (*p* < 0.05). The interaction between these factors was also statistically significant (*p* < 0.05). The results showed that the retention force of the treated groups was significantly improved compared to the untreated group, with the SB+AD group exhibiting the highest retention force, followed by the SB group and then the AD group. Thermal cycling did not affect the retention force in the NT, SB, and SB+AD groups. These findings suggest that the combination of mechanical and chemical surface treatments is the most effective method for improving the retention force between PEEK posts and resin composites for core build-up. Furthermore, appropriate surface treatment of PEEK posts may influence their long-term durability.

## 1. Introduction

Teeth treated with endodontic procedures are structurally weakened due to the loss of pulp and root canal treatment [1]. In particular, when the coronal structure is extensively lost, achieving sufficient retention of the restoration to the remaining dentin is often difficult [2,3]. To ensure the long-term survival of such teeth, performing core build-up procedures is recommended [2]. The design of the core build-up and the materials used are known to significantly impact the survival rate of endodontically treated teeth and the stability of subsequent crown restorations [4,5]. Modern core build-up methods are broadly classified into indirect methods, where the post and core are fabricated as a single unit using casting or CAD/CAM, and direct methods, which combine prefabricated posts with resin composite cores. The former method offers advantages such as ensuring precise adaptation to the individual root canal structure of the patient. However, the metals used in casting methods have significantly different elastic moduli compared to teeth, which increases the risk of severe root fractures [6,7]. Additionally, the increased number of visits and additional technical procedures raise concerns about higher costs [8]. In CAD/CAM methods, various materials, such as zirconia and hybrid ceramics, have been used [9], which reportedly reduce the risk of root fractures mentioned earlier [10]. On the other hand, prefabricated posts used in direct methods have a larger cement thickness between the post and the root canal dentin compared to customized posts. This can lead to the formation of pores and microcracks within the cement, causing stress concentration and fractures at the interface [11]. However, previous studies have reported no significant difference in the survival rates of prefabricated posts compared to customized posts [12]. Furthermore, prefabricated posts are adaptable to root canals with undercuts and are easier to remove during retreatment [13], suggesting their continued clinical application in the future. Currently, prefabricated posts are available in stainless steel, titanium, ceramic, and glass fiber-reinforced composite (FRC) materials. Among these, FRC posts combined with resin composite cores fabricated using the direct method are widely used in clinical practice [14]. This is because FRC posts have a lower risk of root fractures [15,16] and provide better aesthetics compared to metal posts. However, FRC posts face challenges in adhesion to the root canal, and post detachment has been reported [15,16]. Additionally, while the elastic modulus of FRC posts (45.7–53.8 GPa) [17] is lower than that of metal posts, it is still more than twice that of dentin (20–25 GPa) [18], which is of concern. In studies evaluating long-term clinical survival rates, it has been reported that the differences in material properties between metal posts and FRC posts do not affect long-term survival rates [12,19]. On the other hand, it has also been reported that differences in elastic modulus increase the risk of root fracture and irreversible failure when using metal posts compared to FRC posts [20,21]. Consequently, post materials that possess excellent esthetics, appropriate mechanical properties, and superior long-term survival rates are highly desired in clinical practice.

Polyetheretherketone (PEEK) is a semi-crystalline thermoplastic polymer with a low elastic modulus and similar flexural strength compared to dentin [22,23]. Furthermore, PEEK is aesthetically superior to metal and has shock-absorbing properties [23]. Due to these advantages, the application of PEEK has been reported across various dental fields, including crown restorative materials, implantology, orthodontics, and prosthodontics [24]. Furthermore, recent studies have increasingly investigated the potential application of PEEK as a post material [24,25,26,27,28]. In silico and in vitro studies have suggested that PEEK posts may be advantageous over other dental posts in terms of stress distribution. Additionally, the combination of prefabricated PEEK posts and resin composite cores has been suggested to be more effective in preventing root fractures compared to the combination of prefabricated FRC posts and resin composite cores [29]. On the other hand, PEEK materials exhibit disadvantages such as chemical inertness, low surface energy, and resistance to surface modification, which hinders reliable bonding to tooth structure or other dental materials [30]. Therefore, to ensure the long-term stability of PEEK posts, it is necessary to consider adhesion between the post and dentin, as well as between the post and resin composite core. Previous studies have evaluated the adhesion between dentin and PEEK posts [4], as well as the adhesion between PEEK and resin composite materials for veneering [31]. These studies have reported that PEEK with surface treatments exhibits certain adhesion properties to dentin and resin composite. However, these previous studies primarily evaluated adhesion to either veneering materials or dentin. Furthermore, while only a few reports have investigated the bond strength between prefabricated PEEK posts and resin composite cores fabricated using the direct method [32], research simulating their long-term clinical use in the oral cavity is still lacking.

The purpose of this study was to investigate the retention force of PEEK posts with different surface treatments and resin composites for core build-up, and the effect of thermal cycling on the retention force. The null hypotheses were that: (1) There would be no significant difference in the retention force between PEEK posts and resin composites for core build-up among the different surface treatments (no treatment, mechanical treatment, chemical treatment, and a combination of mechanical and chemical treatments). (2) Thermal cycling would have no effect on the retention force between the surface-treated PEEK posts and the resin composites.

## 2. Materials and Methods

### 2.1. Preparation of Specimens for the Pull-Out Test

The retention force between PEEK posts and resin composites for core build-up was evaluated using a pull-out test. Table 1 shows the PEEK material, surface treatment agents, and resin composite for core build-up used in this study. The PEEK posts used for the pull-out test were milled from CAD/CAM PEEK disks (Shofu PEEK, SHOFU, Kyoto, Japan) and cut into cylindrical shapes with an initial diameter of 1.6 mm and a length of 22 mm. The final length was adjusted to 20 mm.

**Figure 1:**
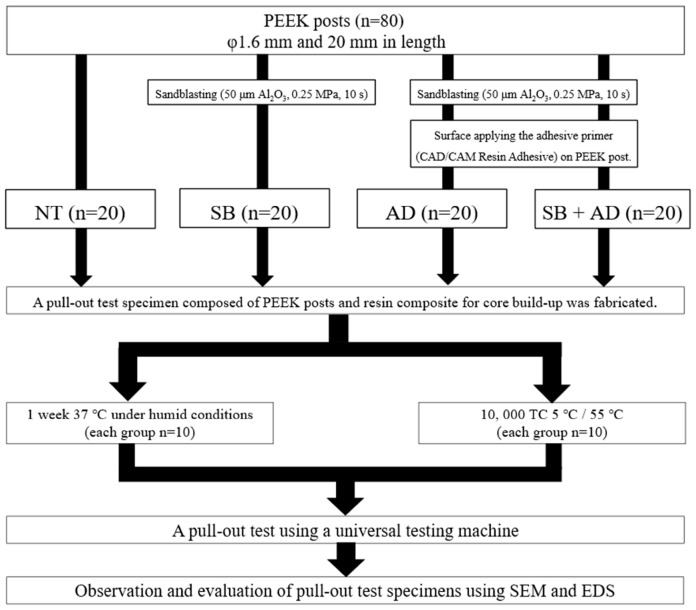
Study design. Study groups: Untreated (NT); Mechanical treatment: sandblasting with 50 μm Al_2_O_3_ particles (SB); Chemical treatment: conditioning using adhesive primer (AD); Combined mechanical and chemical treatment: sandblasting with 50 μm Al_2_O_3_ particles plus conditioning using adhesive primer (SB+AD).

The prepared PEEK posts were classified into four groups based on the surface treatment method and the presence or absence of thermal cycling tests. Figure 1 shows the flowchart of the experimental procedure. The groups were as follows: 1. untreated group (NT); 2. mechanically treated group (SB) with sandblasting using 50 μm Al_2_O_3_ at 0.25 MPa for 10 s from a distance of 10 mm at a 45° angle (HiBlaster Ovaljet, SHOFU, Kyoto, Japan). Subsequently, the treated specimens were cleaned in an ultrasonic bath with distilled water for 15 min and allowed to air-dry; 3. chemically treated group (AD) with primer application (CAD/CAM Resin Adhesive, SHOFU, Kyoto, Japan) followed by air blowing at a low pressure and light curing for 10 s using a light-curing unit (Pencure, Morita, Osaka, Japan); and 4. a group with combined mechanical and chemical treatments (SB+AD). Each group consisted of 20 specimens.

**Figure 2:**
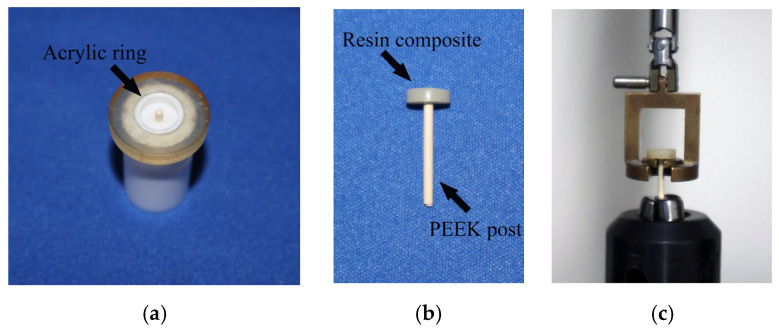
Fabrication of specimens for the pull-out test. (**a**) PEEK post and acrylic ring placed in mold; (**b**) PEEK post and resin composite specimen for pull-out test; (**c**) Specimen during pull-out test.

Preparation of the pull-out test specimens was conducted based on previous studies [33]. Briefly, a mold was prepared to place the treated PEEK post at the center of the specimen with 2 mm of the post exposed above the surface. An acrylic ring (inner diameter: 8 mm, height: 2 mm) was then attached to the mold (Figure 2a). The resin composite for core build-up (Clearfil DCcore Automix, Kuraray Noritake Dental, Tokyo, Japan) was filled into the rings. After filling, a glass plate was pressed against the acrylic ring, and the resin was light-cured at four points at a 90-degree angle for 10 s each using a light-curing unit. After curing, the specimens were removed from the mold and light-cured again at four points at a 90-degree angle for 10 s each from the bottom. These specimens were used for the pull-out test (Figure 2b).

### 2.2. Thermal Cycling

Half of the specimens in each group were stored in a humid environment at 37 °C for one week (*n* = 10 for each group) (TC0). The remaining half were subjected to 10,000 thermal cycles in an automatic thermal cycling unit (Thermocycling HA-K178, Tokyo Giken, Tokyo, Japan) (*n* = 10 for each group) (TC10,000). One thermal cycle consisted of alternate immersion in thermostatically controlled water baths at 5 °C and 55 °C for 30 s each [34]. These two temperatures were selected to approximate the minimum and maximum temperatures experienced in the oral cavity [34,35]. Based on previous literature, the dwell time in each bath was set to 30 s, with a transfer time of 3–5 s between the baths [34].

### 2.3. Pull-Out Test

For the pull-out test, specimen from each group were mounted on a universal testing machine (EZ-Graph, Shimadzu, Kyoto, Japan), and the post portion was gripped with a three-jaw chuck fixture and pulled out vertically from the composite resin portion at a crosshead speed of 0.5 mm/min (Figure 2c). The maximum fracture load obtained was defined as the retention force between the PEEK posts and resin composites.

### 2.4. Observation After Pull-Out Test

After the pull-out test, the surface of the specimens was examined using a stereomicroscope (Stemi508, Carl Zeiss, Jena, Germany) at 40× magnification and a scanning electron microscope (SEM: TM4000Plus II, Hitachi High Tech, Tokyo, Japan). For SEM analysis, the setting conditions were an acceleration voltage of 15.0 kV. The fracture modes were categorized into two types: adhesive failure (no resin composite adhered to the PEEK post) and mixed failure (combination of adhesive failure at the PEEK-resin composite interface and cohesive failure within the resin composite). Additionally, three specimens from each group were selected after the pull-out test, and the surface elements were analyzed using energy dispersive X-ray spectroscopy (EDS).

### 2.5. Statistical Analysis

The retention force obtained from the pull-out test was analyzed using statistical software (SPSS version 25, IBM, Armonk, NY, USA). Mean retention force values among the groups were statistically processed using two-way ANOVA and Tukey’s multiple comparison test. Furthermore, the effect of thermal cycling on the retention force within each group was examined using Student’s *t*-test. The significance level was set at *p* < 0.05.

## 3. Results

### 3.1. Retention Force Between PEEK Posts and Resin Composites

**Figure 3:**
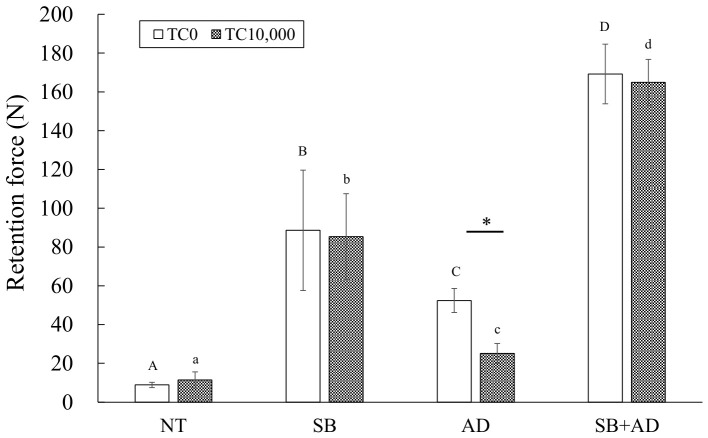
Mean and standard deviations of the retention force of each surface treatment. Different uppercase and lowercase letters represent statistically significant differences between the non-thermal cycling (TC0) and thermal cycling (TC10,000) groups, respectively. Asterisks indicate statistically significant differences among the retention force for the TC0 and TC10,000 groups for the same surface treatment.

The results of two-way ANOVA conducted on the retention force data are shown in Table 2. Both the “surface treatment” factor and the “thermal cycling” factor had statistically significant effects on the retention force (*p* < 0.05). The interaction between these factors was also statistically significant (*p* < 0.05). Figure 3 shows the retention force between PEEK posts and resin composites. The mean retention forces were 9 ± 1 N for the NT group (TC0) and 11 ± 4 N for the NT group (TC10,000); 89 ± 31 N (TC0) and 85 ± 22 N (TC10,000) for the SB group; 52 ± 6 N (TC0) and 25 ± 5 N (TC10,000) for the AD group; and 169 ± 15 N (TC0) and 165 ± 12 N (TC10,000) for the SB+AD group. Significant differences in the retention force were observed among all surface treatments, regardless of the presence or absence of thermal cycling. The retention force was highest in the SB+AD group, followed by the SB group, AD group and the NT group, in that order. Additionally, the AD group showed a significant decrease in the retention force after thermal cycling, while the other groups were not affected by thermal cycling.

### 3.2. Observation After Pull-Out Test

**Figure 4:**
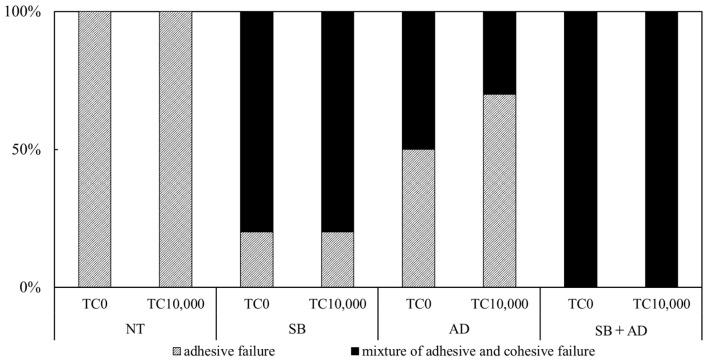
Fracture mode ratio after the pull-out test.

**Figure 5:**
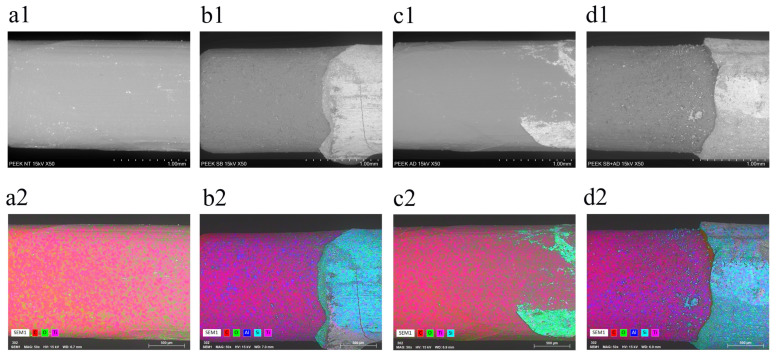
PEEK post surface after the pull-out test without thermal cycling. The upper section of the figure shows SEM images, while the lower section shows mapping images. NT (**a1**,**a2**); SB (**b1**,**b2**); AD (**c1**,**c2**); SB+AD (**d1**,**d2**).

**Figure 6:**
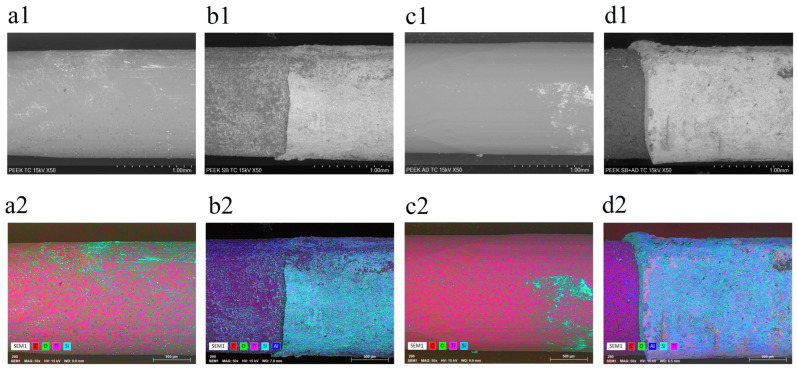
PEEK post surface after the pull-out test with thermal cycling. The upper section of the figure shows SEM images, while the lower section shows mapping images. NT (**a1**,**a2**); SB (**b1**,**b2**); AD (**c1**,**c2**); SB+AD (**d1**,**d2**).

Figure 4 shows the ratio of fracture modes after the pull-out test. In the NT group, 100% of the specimens exhibited adhesive failure, regardless of the thermal cycling condition (TC0 or TC10,000). In the SB group, mixed failure was observed in 80% of the specimens, regardless of thermal cycling. In contrast, the AD group demonstrated a 50% adhesive failure rate at TC0, which increased to 70% at TC10,000. In the SB+AD group, 100% mixed failure was consistently observed across all specimens. Overall, these results indicate a tendency wherein higher retention forces obtained from the pull-out test are associated with an increased proportion of mixed failures. Typical SEM images and EDS element mapping of the surfaces of specimens in each PEEK-post group after the pull-out test are shown in Figure 5 for TC0 and in Figure 6 for TC10,000. SEM observation revealed that the NT group exhibited a smooth PEEK post surface regardless of thermal cycling. In the SB and SB+AD groups, roughened surfaces induced by sandblasting were observed, with abundant remnants detected across all specimens. Conversely, the AD group showed a mixture of specimens with and without remnants; in particular, a trend toward a minimal amount of remnants was observed in the specimens after thermal cycling. Notably, no fractures of the PEEK posts were observed in any of the specimens.

EDS analysis predominantly detected carbon, oxygen, and titanium on the PEEK surfaces. In the sandblasted specimens, aluminum derived from alumina was detected, while silicon was detected within the remnants. Since silicon is a constituent element of the filler in resin composite, this finding indicates that the remnants originated from the resin composite. These results demonstrate that appropriate pretreatments were performed in the SB and SB+AD groups, thereby maintaining the retention between the PEEK and the resin composite irrespective of thermal cycling. On the other hand, the AD group exhibited a decrease in retentive force due to the influence of thermal cycling.

## 4. Discussion

The purpose of this study was to compare and evaluate the retention force between PEEK posts with mechanical and chemical surface treatments and resin composites for core build-up, as well as the effects of thermal cycling on the retention force. The results showed that the retention force of the treated groups was significantly higher than that of the untreated group. Regarding the effects of thermal cycling, the group with only chemical treatment showed a significant decrease in the retention force, while the other groups were not affected by thermal cycling. Therefore, (1) the first null hypothesis, that there would be no significant difference in the retention force between PEEK posts and the resin composites among the different surface treatments was rejected. On the other hand, (2) the second null hypothesis, that thermal cycling would have no effect on the retention force between the PEEK posts and the resin composites was partially accepted. Prefabricated posts have various adhesive interfaces with core materials and root dentin. In vitro studies have evaluated the bond strength of these interfaces using shear tests, microtensile tests, pull-out tests, and push-out tests [36]. Recent studies have increasingly used push-out tests for evaluation due to their ability to assess local differences in bond strength [32,36]. However, in this study, the retention force between PEEK posts and resin composites for core build-up was evaluated using a pull-out test. This is because the study focused solely on the overall retention force of the resin composite bonded to the post, rather than local differences in bond strength. Additionally, push-out tests require cutting the specimen during preparation, which may damage or fracture the specimen [37].

### 4.1. Surface Treatment of PEEK

One of the factors affecting the durability of resin composite core restorations is the strong bond between the core material and the post material [38]. Appropriate surface treatment of the post can improve the bond strength with the resin composite core [32,33,38,39]. However, the PEEK material used in this study is chemically inert, has low surface energy, and is resistant to surface modification [30]. Therefore, it is crucial to consider appropriate surface treatments for PEEK to form a strong and durable bond with resin materials. Effective surface treatments for PEEK include sulfuric acid treatment [4,32,40,41], which has been reported to improve mechanical interlocking by increasing surface roughness through the formation of a porous surface and enhancing chemical bonding by forming sulfonic groups on the PEEK surface. However, using sulfuric acid for chairside treatment is challenging due to its toxicity and the risk of oral tissue damage. On the other hand, sandblasting with alumina particles is widely used in clinical practice to enhance mechanical interlocking. It has been reported that sandblasting increases the surface roughness of PEEK, thereby improving its bond strength [30,31,40,41]. In this study, SEM observations and EDS results confirmed the formation of sandblasted surfaces on PEEK. Furthermore, the retention force between sandblasted PEEK and resin composite was significantly improved compared to untreated PEEK, regardless of thermal cycling, suggesting that sandblasting is effective for surface treatment of PEEK posts. Chemical surface treatment using methyl methacrylate (MMA)-based primers has also been recommended for PEEK. Although the mechanism by which MMA monomers enhance adhesion to PEEK is not yet fully understood, Hata et al. suggested that a semi-interpenetrating polymer network structure forms at the interface between PEEK and MMA monomers, contributing to improved adhesion [42]. This structure consists of polymer blends at the molecular level, where MMA molecules penetrate the PEEK polymer chains and polymerize into linear polymers, creating mechanical interlocking at the interface. Huang et al. reported that MMA improves the wettability of the PEEK surface, which they attributed to the formation of polar groups (e.g., carbonyl groups) on the PEEK surface [43]. Additionally, the primer used in this study contained urethane dimethacrylate (UDMA), which enhances fluidity, allowing better penetration into the PEEK surface. UDMA also improves physical properties through its urethane bonds, further enhancing adhesion [43]. These effects likely contributed to the retention force between PEEK and resin composites for core build-up in this study. The results of this study suggest that the combination of mechanical and chemical surface treatments is most effective in improving the retention force between PEEK posts and resin composites for core build-up. This is likely due to the optimal adhesion performance achieved by the deep penetration of low-viscosity MMA and UDMA-containing monomers into the rough surface created by appropriate sandblasting. Furthermore, previous studies have shown that combining mechanical and chemical treatments for polyaryl ether polymers like PEEK yields synergistic effects compared to using either treatment alone [38,43,44,45], supporting the findings of this study. In contrast, the retention force significantly decreased after thermal cycling in the group receiving only chemical pretreatment. This indicates that the primer was the most affected by thermal cycling among the materials used in this study. It has been previously suggested that thermal expansion induced by thermal cycling promotes water penetration into the bonding interface, resulting in resin failure [46]. This phenomenon is particularly attributed to water sorption and monomer elution from UDMA [46]. The present study exhibited a trend consistent with this existing knowledge. However, as other factors—such as stress induced by mismatched coefficients of thermal expansion between the materials or hydrolysis at the bonding interface—may also play a role, further investigation is necessary.

### 4.2. Effects of Thermal Cycling

The results of this study indicate that, except for the group with only chemical treatment, the effects of thermal cycling on the retention force between PEEK posts and resin composites for core build-up were minimal. PEEK possesses highly stable chemical and physical properties due to the presence of ketone and aryl groups [47,48], resulting in lower solubility and water absorption compared to other dental polymers [47,48,49]. Additionally, PEEK has lower thermal conductivity compared to metals and fiber posts, which minimizes thermal stress on adhesive interfaces and suggests its potential for long-term use [25].

In this study, the thermal cycling test involved 10,000 cycles, equivalent to approximately one year of use in the oral cavity [33,50]. Furthermore, in the clinical oral environment, it is necessary to consider not only degradation due to temperature changes but also stresses generated by mastication and occlusal forces. Altitinchi et al. investigated the fatigue resistance of metal, zirconia, and fiber posts by combining thermal cycling with a mastication simulation [51]. They reported that metal and zirconia posts exhibited significantly higher fatigue resistance than fiber posts. They discussed that this outcome was due to the high-strength post materials being better able to withstand the prolonged stress of 1.2 million cycles and the relatively large occlusal forces equivalent to those on premolars. Conversely, studies evaluating fracture resistance under static loading tests have shown that PEEK and other low-elastic-modulus materials exhibit excellent fracture resistance [29]. Therefore, further evaluations involving a greater number of thermal cycles and mastication simulations are required to fully elucidate the long-term stability of PEEK posts and resin composite cores.

### 4.3. Retention Force Between PEEK and Resin Composites for Core Build-Up

In this study, the maximum retention force between PEEK posts and resin composites for core build-up was achieved with the combination of mechanical and chemical surface treatments, with an average value of 169 ± 15 N. Under the same experimental conditions, studies evaluating the retention force between FRC posts and resin composites reported values ranging from 250 N to 310 N [33]. Therefore, in terms of the retention force with resin composites for core build-up, PEEK posts may be inferior to FRC posts. Furthermore, Singh et al. reported that the clinically required retention force is approximately 200 N or higher under masticatory loading [52]. Therefore, within the limitations of the present study, it was suggested that the retention force between the PEEK post and the resin composites obtained with the tested surface treatments might fall below the clinically acceptable threshold.

On the other hand, although the experimental methods and surface treatment conditions differed, studies evaluating the bond strength between resin composites and PEEK or FRC posts using push-out tests have reported that sandblasted PEEK posts exceed the bond strength of FRC posts [53]. Therefore, further research is needed to determine the optimal surface treatment method for achieving long-term retention between PEEK posts and resin composites for core build-up. Another factor to consider is the light intensity of the curing lamp used in this study, which was approximately 650 mW/cm^2^. Considering that contemporary LED light-curing units typically feature an output intensity of around 1000 mW/cm^2^ [54], this lower intensity could potentially affect the mechanical properties of the core build-up resin composite. On the other hand, it has been reported that the minimum acceptable irradiance required for clinical success is 300 mW/cm^2^ [54]. Moreover, since the core build-up resin composite used in this study is a dual-cure material, this light intensity of 650 mW/cm^2^ is considered sufficient to achieve adequate initial polymerization.

### 4.4. Limitation

As a limitation of this study, a pull-out test was utilized to focus solely on the retention force between the PEEK post and the composite resin core material. However, the retention of the post to the root canal dentin is as crucially important as its bond to the core material. Therefore, future studies need to incorporate a push-out test to evaluate site-specific local variations in bond strength, alongside exploring the fabrication of integrated specimens that encompass the core, post, and dentin complex. Moreover, aging in the oral cavity was evaluated exclusively through thermal cycling in the present study. To more accurately simulate clinical conditions, the mechanical stresses induced by masticatory and occlusal forces should be considered, necessitating the combined application of chewing simulation alongside thermal cycling. Additionally, the 10,000 cycles employed in this study correspond to approximately one year of intraoral use. To investigate the long-term behavior further, a greater number of cycles should be examined in future studies.

## 5. Conclusions

1. The retention force between PEEK posts and resin composites for core build-up varies significantly depending on the surface treatment method. The group with combined mechanical surface treatment (sandblasting) and chemical surface treatment (primer application) shows the highest retention force.
2. After conducting thermal cycling tests simulating one year of use in the oral cavity, none of the groups was affected by thermal cycling, except for the group with only chemical surface treatment.
3. Within the limitations of this study, the retention force of the PEEK post with a 2 mm bonding interface was 169 ± 15 N, which was lower than that of the FRC post.

## Figures and Tables

**Table 1:** Materials used in this study.

Material Type	Product (Lot No.)	Main Components	Manufacturer
Polye there therke tone (PEEK)	SHOFU PEEK (23010602)	Poly-ether-ether-ketone	Shofu, Kyoto, Japan
Resin composite for core build-up	Clearfil DCcore Automix (BA0525)	Bis-GMA, TEGDMA, Hydrophobic aromatic dimethacrylate, silanated barium glass filler, silanated silica, CQ, BPO, accelerators	Kuraray Noritake Dental, Tokyo, Japan
Adhesive primer	CAD/CAM Resin Adhesive (092201)	UDMA, MMA, acetone, initiators and others	Shofu, Kyoto, Japan

TEGDMA: Triethyleneglycol dimethacrylate; CQ: Camphorquinone; BPO: Benzoyl peroxide; UDMA: Urethane dimethacrylate; MMA: Methyl methacrylate.

**Table 2:** Results of two-way ANOVA performed for evaluation of the retention force between PEEK and each surface treatment, both with and without thermal cycling.

	Sum of Square	D*f*	*F* Value	*p* Value
Surface treatment	282,692.20	3	394.090	<0.05
Thermal cycling	1305.11	1	5.458	<0.05
Surface treatment × thermocycling	2596.87	3	3.619	<0.05

## Data Availability

The original contributions presented in this study are included in the article. Further inquiries can be directed to the corresponding author.
